# The Emergence and Spread of Novel SARS-CoV-2 Variants

**DOI:** 10.3389/fpubh.2021.696664

**Published:** 2021-08-02

**Authors:** Huaimin Yi, Jin Wang, Jiong Wang, Yuying Lu, Yali Zhang, Ruihao Peng, Jiahai Lu, Zeliang Chen

**Affiliations:** ^1^NMPA Key Laboratory for Quality Monitoring and Evaluation of Vaccines and Biological Products, One Health Center of Excellence for Research and Training, School of Public Health, Sun Yat-sen University, Guangzhou, China; ^2^School of Public Health, Southern Medical University, Guangzhou, China; ^3^Key Laboratory of Livestock Infectious Diseases in Northeast China, Ministry of Education, Shenyang Agricultural University, Shenyang, China

**Keywords:** SARS-CoV-2, variants, spike protein, mutations, characteristics, epidemiology

## Abstract

Since severe acute respiratory syndrome coronavirus-2 (SARS-CoV-2) began to spread in late 2019, laboratories around the world have widely used whole genome sequencing (WGS) to continuously monitor the changes in the viral genes and discovered multiple subtypes or branches evolved from SARS-CoV-2. Recently, several novel SARS-CoV-2 variants have been found to be more transmissible. They may affect the immune response caused by vaccines and natural infections and reduce the sensitivity to neutralizing antibodies. We analyze the distribution characteristics of prevalent SARS-CoV-2 variants and the frequency of mutant sites based on the data available from GISAID and PANGO by R 4.0.2 and ArcGIS 10.2. Our analysis suggests that B.1.1.7, B.1.351, and P.1 are more easily spreading than other variants, and the key mutations of S protein, including N501Y, E484K, and K417N/T, have high mutant frequencies, which may have become the main genotypes for the spread of SARS-CoV-2.

## Introduction

The three highly transmissible pathogens, including severe acute respiratory syndrome coronavirus (SARS-CoV), Middle East respiratory syndrome coronavirus (MERS-CoV), and severe acute respiratory syndrome coronavirus-2 (SARS-CoV-2), which have emerged in humans over the past 20 years, are coronavirus species (1, 2). No matter the number of infected people, the spatial range of the epidemic area and the duration of the epidemic, SARS-CoV-2 has overwhelmingly surpassed SARS-CoV and MERS-CoV (3). Moreover, the coronavirus disease 2019 (COVID-19) caused by SARS-CoV-2 is so highly contagious that it has spread rapidly around the world, posing a huge threat to global public health.

The genome of coronaviruses is a linear single-stranded, positive-sense RNA genome. The error rate of RNA replication (about 10^−4^ per year per site) is generally greater than that of DNA (about 10^−5^) (4, 5). Compared with DNA polymerase, RNA polymerase that catalyzes the replication of RNA molecules has no proofreading capabilities and no mechanism for post-replication mismatch repair (6). Therefore, the potential for RNA mutation is high. Different from general RNA viruses, some studies (7, 8) have found that coronaviruses can provide the proofreading capabilities to maintain large RNA genomes without accumulating detrimental mutations, while some researchers (9, 10) think, compared with other single-stranded RNA (ssRNA) viruses, the estimated mutation rates in coronaviruses are at least moderate. In other words, the coronavirus genome allows additional plasticity for genome modification through mutation and recombination, thereby, increasing the possibility of intraspecies variation and interspecies transmission (host switching/jumping). So far, SARS-CoV-2 accumulates mutations at a rate of about one to two changes per month (11).

The genome of SARS-CoV-2 encodes both six functional open reading frames (ORFs) and four structural proteins—spike (S), membrane (M), envelope (E), and nucleocapsid (N) (12). Among them, the S proteins, one of the major structural proteins, form homotrimers protruding on the surface of the virus, which is crucial for the virus to enter the cell. S protein is cleaved by furin-like proteases in host cells into functional subunits S1 and S2, which are respectively, responsible for determining the host range and cell tropism of the virus and driving fusion between the virus and the host cell (13–15). Besides, S1 contains a receptor-binding domain (RBD) that binds to angiotensin-converting enzyme 2 (ACE2) to initiate the entry of the virus into cells (16). Therefore, S protein is considered a key molecular target for vaccine design, therapeutic antibodies, and diagnostic methods.

Thanks to WGS technology, SARS-CoV-2 variants have been discovered in many regions of the world. The rapid evolution of the SARS-CoV-2 variant whose mutations occurred in the S gene region has raised concerns that these mutations may alter the amino acid sequence of neutralizing antibody epitopes, thereby affecting the effectiveness of therapeutic antibodies and vaccines.

In this study, we analyze the distribution characteristics of prevalent SARS-CoV-2 variants in the world so as to provide evidence for Institutes of Health to quickly grasp epidemic transmission among countries and facilitate the formulation of prevention timely. Moreover, we integrate the frequency of mutations at different sites on S protein and discover the key sites of mutant hotspots in time. It is helpful to timely understand the impact of variants on infection, diagnosis, and treatment, which has certain significance for guiding international public health decision-making.

## Methods

### Data Collection

All available SARS-CoV-2 data were downloaded from the GISAID (https://www.gisaid.org/hcov19-mutation-dashboard/) and PANGO (https://cov-lineages.org/global_report.html). All receptor binding site changes in S protein found by genome sequencing are reported in the Table on the GISAID (GISAID automatically updates this table daily; the data collection date is as of March 3, 2021), namely, list all RBS changes. The data of the number of variant sequences and total sequences since first variant sequence are listed in a complete table on the PANGO (data collection date is as of April 4, 2021).

### Statistical Analysis

We performed a descriptive epidemiology analysis. R version 4.0.2 was used for statistical analysis, and ArcGIS 10.2 software was used for mapping. Data were presented as frequency and percentage.

## Results

### The Characteristics of New Variants

**B.1.1.7:** It is a new variant under investigation (VUI 202012/01 or variant of concern, VOC 202012/01) of the 501Y lineage, based on the B.1.1.7 lineage of PANGO lineage, GISAID clade GR/501Y.V1, Nextstrain clade 20I/501Y.V1 (https://nextstrain.org/). The first sample to identify the virus was found in a retrospective study in the UK on September 20, 2020 (17, 18). B.1.1.7 has an unusually large number of mutations in a single cluster, including 14 non-synonymous mutations (amino acid changes), six synonymous mutations (amino acid does not change), and 4 deletions (19).

Three mutations in S gene of these mutations have potential biological effects: First, mutation N501Y is located in the receptor-binding motif (RBM), one of the six key contact residues within the receptor-binding domain (RBD), and has been identified to enhance the affinity of the virus to ACE2 (20, 21). Second, 69–70 deletion may lead to conformational changes of S protein that it is structural changes, which is conducive to the escape of the virus from the immune response of the host (22). Third, mutation P681H is located near the insertion sites of four amino acids, connecting S1 and S2 subunits in S protein, in other words, adjacent to the furin cleavage site, which may cause S protein to be more easily cleaved by the protease, thereby, enhancing its affinity with the ACE2 receptor and promoting the virus to enter respiratory epithelial cells (23–25).

**B.1.351:** The South African government held a press conference to announce the first discovery of variant B.1.351 (also known as “GH/501Y.V2” or “20H/501Y.V2”) on December 18, 2020, dating back to early October 2020, and, now, B.1.351 is one of the most popular variants in the world (26, 27). There are three mutations, N501Y, K417N, and E484K, in the RBD of S protein. E484K is located in RBM and directly contacts specific ACE2 residues. There is evidence that mutation E484K may affect the neutralization of therapeutic antibodies (28, 29). Although, K417N site does not combine with ACE2, it is an epitope of neutralizing antibody-like E484K, and so it may be selected to evade humoral immune reaction (29, 30).

**P.1:** The P.1 lineage (also known as “GR/501Y.V3” or “20J/501Y.V3”), a descendant of B.1.1.28, is first reported in Japanese travelers returning from Amazon, Brazil in January 2021, and the first sequence was noted in GISAID from Brazil in December 2020 (31, 32). It has 17 unique amino acid changes, 3 deletions, 4 synonymous mutations, and one 4nt insertion, including three mutations in the RBD of S protein: K417T, E484K, N501Y (32, 33). The physiological function of K417T is similar to K417N.

**B.1.525:** Public Health England (PHE) found a variant B.1.525 (VUI202102/03) from 38 COVID-19 cases on February 16 (34). The mutations Q52R, E484K, Q677H, and F888 are in S protein (35). As of March 8, 2021, the variant has been detected in 26 countries (36).

**CAL.20 C:** The variants (including lineage B.1.429 and B.1.427) were first discovered in Southern California, USA in July 2020, and gradually became the main local epidemic strain. The mutations of S protein include S13I, W152C, and L452R (37, 38). L452R, located in RBM, may increase infectivity by enhancing the binding of S protein to ACE2 receptor and evading neutralizing antibodies and has evolved independently in multiple lineages (39).

**COH.20G:** In late December, two variant strains of COH.20G/677H and COH.20G/501Y were detected in Columbus, Ohio, USA. The mutation Q677H and N501Y in S protein have been proved to have higher affinity binding to ACE2 (40, 41).

**Cluster 5** (also known as “ΔFVI-spike”): Some researchers found that mutation Y453F in the RBD of S protein of this variant did not reduce existing humoral immunity or affect the neutralization response, but it increased transmissibility due to its enhanced affinity with ACE2 (42).

**B.1.1.207:** This variant, which was detected by sequencing in August 2020, accounted for ~1% of the sequenced viral genome in Nigeria as of late December 2020 (43). It shares mutation P681H with B.1.1.7, which may represent an independent homogeneity of the UK strain (44).

**Figure 1:**
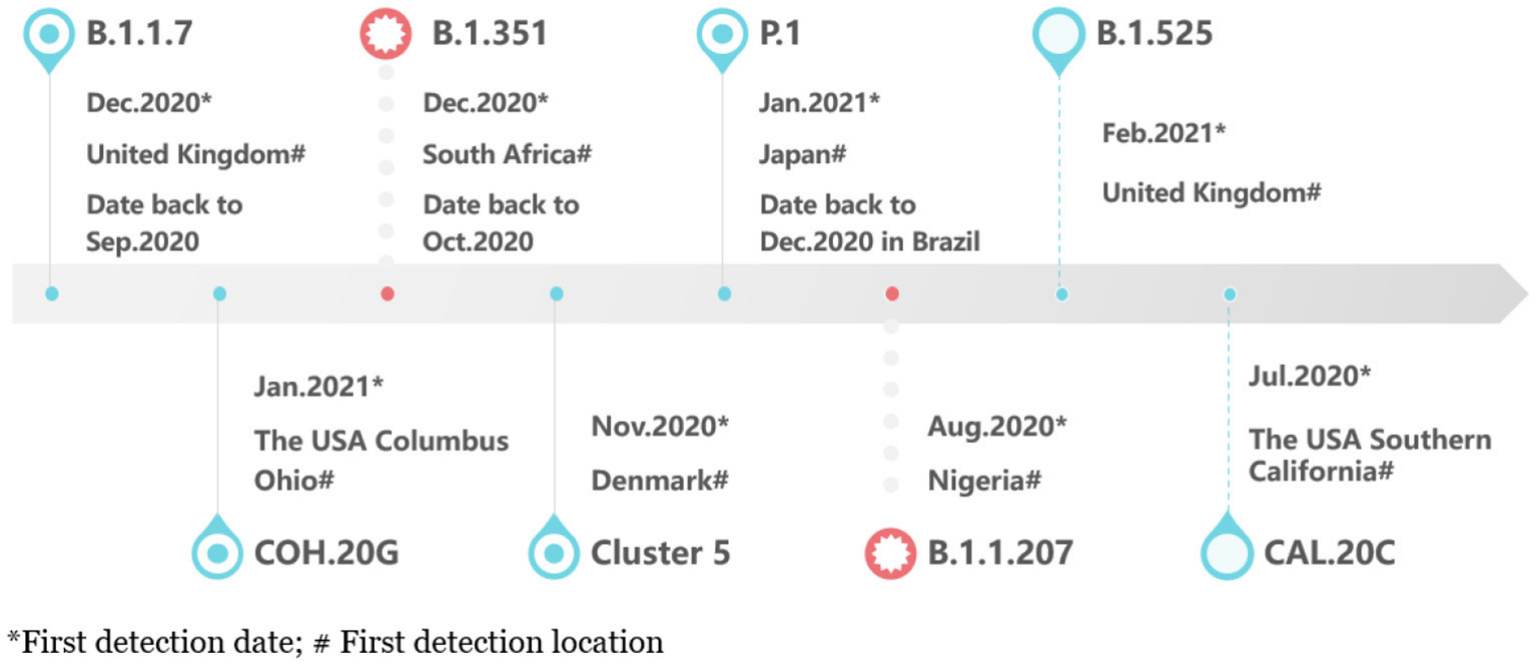
The timeline of new variants.

**Figure 2:**
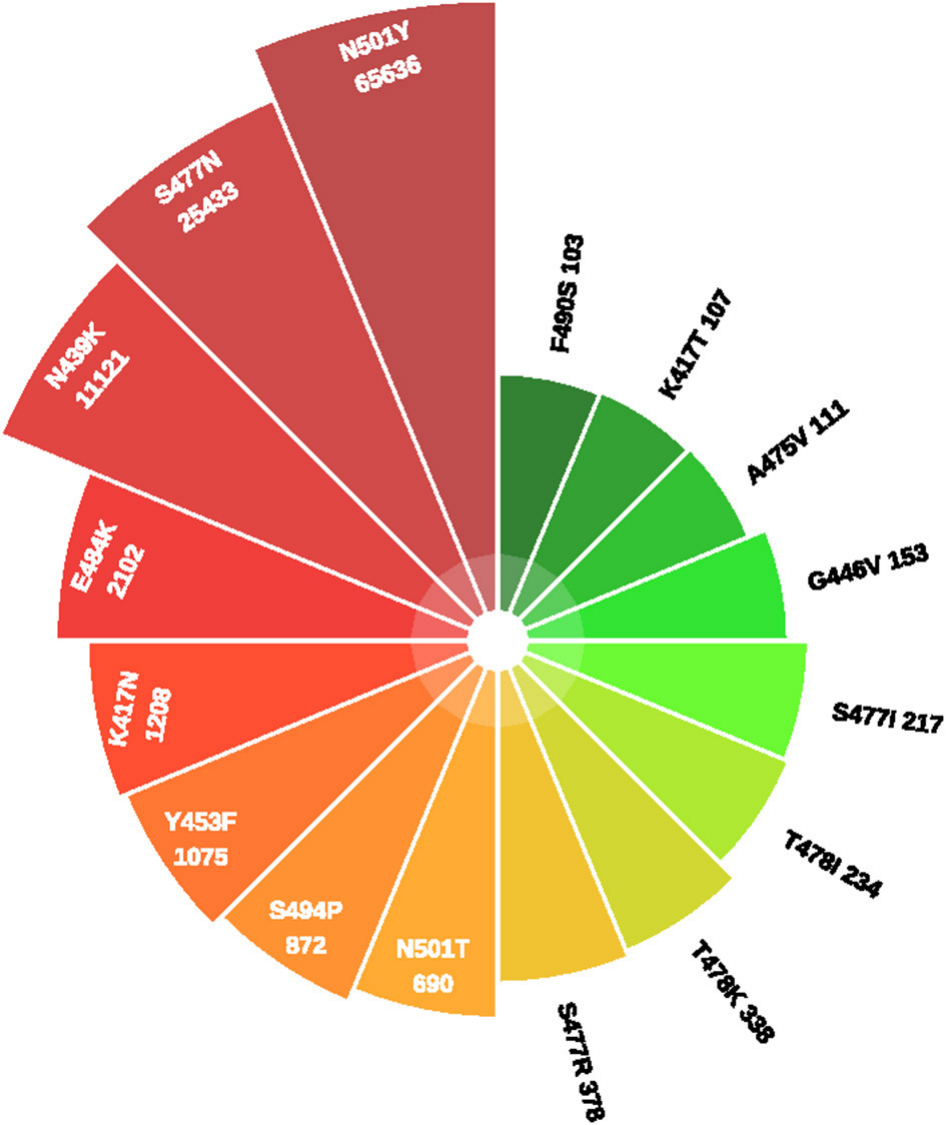
The frequency of mutations in S protein.

Figure 1 showed the timeline of recently international concerned variant strains. Table 1 summarized the amino acid mutant sites of variant strains. The lineages B.1.1.7, B.1.351, P.1, and COH.20G all have mutation N501Y. B.1.351, P.1, and B.1.525 have mutation E484K. Figure 2 lists information of the first 16 sites with high mutant frequencies. According to the GISAID recommendation, the cutoff value is set to 100. The frequencies of N501Y, E484K, K417N, and K417T are respectively, 65,636, 2,102, 1,208, and 107, high on the list. So N501Y, E484K, and K417N/T may have become the main genotypes for the spread of SARS-CoV-2 and may change the structure, properties, and other characteristics of S protein. In addition, Y453F is a high mutation frequency of 1,075, but its role is unclear. In conclusion, these mutations at these sites are worthy of further research.

**Table 1:** New variants of amino acid mutations in the S protein.

**Variants**	**B.1.1.7**	**B.1.351**	**P.1**	**B.1.525**	**CAL.20C**	**COH.20G**	**Cluster 5**	**B.1.1.207**
Spike	**HV 69-70 del**	D80A	L18F	Q52R	L452R	Q677H	**Y453F**	**P681H**
	Y144 deletion	243-245 del	T20N	**E484K**	S13I	**N501Y**		
	**N501Y**	D215G	P26S	Q677H	W152C			
	A570D	**K417N**	D138Y	F888				
	**P681H**	**E484K**	R190S					
	T716I	**N501Y**	**K417T**					
	S982A	A701V	**E484K**					
	D1118H		**N501Y**					
			H655Y					
			T1027I					

*The meaning of the bold values provided in Table 1 is represented by the biologically significant mutation sites on S protein discovered in the current study*.

### The Epidemiology of Main Variants

From an epidemiological perspective, B.1.1.7, B.1.351, and P.1 are more easily spreading than other variants, and they are worse epidemiological situations in the areas where they have recently emerged, resulting in more confirmed COVID-19 cases and putting more pressure on the medical system. There is no evidence showing that these variants cause more serious illness or increase the risk of death (45).

**Figure 3:**
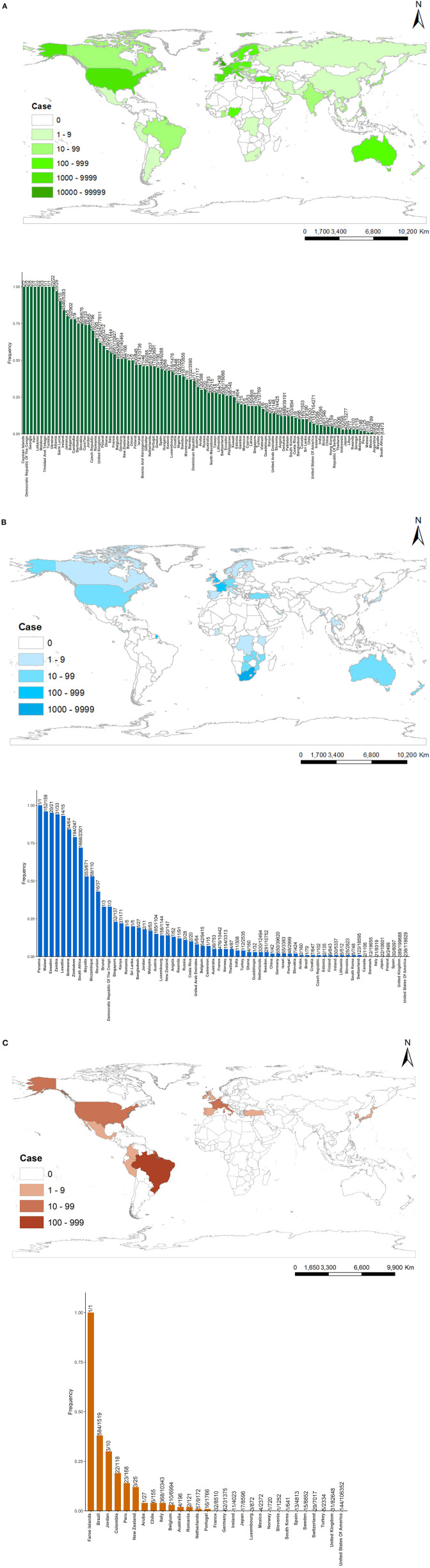
Countries, territories, and areas reporting new variants [**(A)** = B.1.1.7, **(B)** = B.1.351, **(C)** = P.1]. The upper part of **(A–C)** represents the global distribution of variants, and the lower part of **(A–C)** represents the frequency of variants defined that the number of variant sequences divided by the number of total sequences since the first variant sequence.

As of April 4, 2021, 100 countries have identified B.1.1.7 variants, 64 countries have detected B.1.351 variants, and 29 countries have detected P.1 variants (Figure 3). These figures show that B.1.1.7 has the widest distribution in the world and the most cases of infection. B.1.1.7 is mainly distributed in Europe and North America, and the top six countries infected with B.1.1.7 lineages are the UK (173,624), Germany (20,797), the USA (11,514), the Netherlands (6,518), Italy (6,307), and France (5,951). B.1.351 is mainly distributed in Southern Africa, Western Europe, and North America, and the top five countries infected with B.1.351 lineages are South Africa (1,668), Germany (650), Belgium (642), France (476), and UK (389). P.1 is mainly distributed in South America and North America, and the top five countries infected with B.1. 1.28 lineages are Brazil (584), Italy (368), Belgium (210), the USA (144), and Germany (62).

From the perspective of sequencing sample size, developed countries in Europe, America, and Japan have more total sequences since the first variant sequence than other countries, like the UK (277,811), the USA (154,271), Germany (40,464), Denmark (39,191), Switzerland (19,066), Japan (13,277) in B.1.1.7. The UK has the most cases infected with B.1.1.7 in the world, and its total sequences are accounted for 62.50% (173,624/277,811). So, B.1.1.7 becomes the dominant variant in the UK. In the same way, B.1.135 becomes the most variant in South Africa (72.49%, 1,668/2,301), and P.1 becomes the most variant in Brazil (38.45%, 584/1,519).

## Discussion

Some studies indicated that these mutations discovered in these variants cannot only evade the immune response caused by vaccines and natural infections to improve transmissibility but also may reduce sensitivity to neutralizing antibodies (46–50). Moreover, Volz et al. (51) discovered that B.1.1.7 may affect the performance in routine PCR testing analysis for S-gene target failure (SGTF). However, some studies found that current vaccines and therapeutic antibodies were still effective against variant strains (41, 52). In short, the impact of these variants on the effectiveness of currently available vaccines and therapeutic methods is controversial to a certain extent, leading to remain unexplored, so this needs to be further researched (Please refer to Supplementary Table A). Furthermore, there is no evidence showing that the symptoms, severity, duration of the disease caused by variants, and the reinfection rate have changed significantly.

Based on epidemiological analysis, B.1.1.7, B.1.351, and P.1 become the dominant variants in UK, South Africa, and Brazil, respectively, where they first emerged, resulting in more confirmed COVID-19 cases. Moreover, they spread rapidly in the surrounding regions. So, they are more transmissible than preexisting SARS-CoV-2 variants. One of these variants, B.1.1.7, has spread globally, and the number of cases infected with it is the largest of all mutant strains, which shows that it has more advantages in spreading. The reason may be that it has an abnormally large number of genetic mutations, and its mutant sites have many mutations of very high frequency. However, due to differences in the intensity of local genome surveillance, the attention to the introduction of new variants, and the volume of international tourism in different countries, only some samples of confirmed COVID-19 cases have been sequenced, so the possibility of the significant underestimated number cannot be ruled out (53).

Severe acute respiratory syndrome coronavirus 2 naturally mutates and evolves over time, providing it with a selective advantage for the virus to escape immunity, so this virus and its variants may change its pathogenesis, virulence, and transmissibility. Here, we summarize the characteristics of new variants based on available scientific evidence (Please refer to Supplementary Table B). Non-pharmaceutical intervention is still the focus of prevention and control, especially for countries with more cases of mutant strains. Public health strategies, such as social distance, quarantine, wearing masks, and frequent handwashing, are strictly followed to limit the spread of SARS-CoV-2 and protect public health.

## Conclusion

In view of the newly emerging mutant strains, we should continue to focus on the protective effect of the three main mutant strains B.1.1.7, B.1.351, and P.1 on the currently used vaccines and therapeutic antibodies, as well as the impact of the key mutations of S protein on their infectivity, virulence, and antigenicity.

First, all countries are supposed to work together to follow the unified plan of WHO and carry out continuous monitoring of virus sequences and basic scientific research so as to detect the introduction of known variants and the emergence of new variants in time and provide valuable insights into the continuous evolution and epidemiology of these viruses during the pandemic. Second, continue to monitor changes in local transmissibility or severity of infection to identify and evaluate the spread and impact of variants. Third, to ensure effective prevention and control, quarantine should be carried out as soon as possible to control mutant strains in the early stage once adverse mutation occurs.

## Data Availability Statement

The original contributions presented in the study are included in the article/Supplementary Material, further inquiries can be directed to the corresponding author.

## Author Contributions

Data collection and analysis were performed by HY, JinW, and JioW. HY and JinW wrote the manuscript. ZC and JL conceived the idea and performed manuscript review. All authors contributed to the study conception and design, and read and approved the final manuscript.

## Conflict of Interest

The authors declare that the research was conducted in the absence of any commercial or financial relationships that could be construed as a potential conflict of interest.

## Publisher's Note

All claims expressed in this article are solely those of the authors and do not necessarily represent those of their affiliated organizations, or those of the publisher, the editors and the reviewers. Any product that may be evaluated in this article, or claim that may be made by its manufacturer, is not guaranteed or endorsed by the publisher.
