# Supplementary material for: The Emergence and Spread of Novel SARS-CoV-2 Variants
Source: Front Public Health. 2021 Aug 2;9:696664. doi: 10.3389/fpubh.2021.696664 (PMC8364952; doi:10.3389/fpubh.2021.696664)
Supplement: Supplementary file 1 [file Table_1.DOCX]

Supplementary Material

# Supplementary Tables

Table A Effects of variants on vaccines and therapeutic antibodies

| Author | Interventions | The result of research |
| --- | --- | --- |
| Tada T, et al[37] | BNT162b2(Pfizer)  mRNA Vaccine | 1. Neutralized antibodies for B.1.1.7 S protein with titers that were 7-fold greater than convalescent sera.  2. Neutralized antibodies for B.1.351 S protein reduced 3-fold in titer (1:500) than convalescent sera, but still higher than convalescent sera neutralized D614G (1:139).  3. BNT162b2 may maintain protective efficacy against B.1.1.7 and most other variants but that the partial resistance of virus with the B.1.351 S protein.  4. A pseudovirus assay showed that convalescent sera for B.1.1.7, B.1.351, COH.20G/677H, 20A.EU2 Europe and cluster 5. S proteins with only a minor decrease in titer compared to that of the earlier D614G S protein. |
| Wu K, et al[48] | mRNA-1273 (Moderna)  mRNA Vaccine | 1. No significant impact on neutralization against the B.1.1.7 variant was detected following mRNA-1273 vaccination.  2. Pseudoviruses with full B.1.351 mutations reduced 6.4-fold Geometric mean titer, but mRNA-1273 vaccination was still significant neutralization against it. |
| Shen X, et al[43] | mRNA-1273 (Moderna)+  NVX-CoV2373(Novavax)  mRNA Vaccine+  S protein nanoparticle Vaccine | A lentivirus-based pseudovirus assay to show that variant B.1.1.7 remains sensitive to neutralization, albeit at moderately reduced levels (~2-fold), by serum samples from convalescent and vaccinated two vaccines mRNA-1273 and NVX-CoV2373. |
| Starr TN, et al[35] | LY-CoV555+LY-CoV016 cocktail or alone (Bryan Jones and Eli Lilly)  mAb | 1. E484K escapes LY-CoV555 and K417N/T escape LY-CoV016 in B.1.351 and B.1.1.28, L452R mutation in B.1.429 escapes LY-CoV555, and N501Y impacts neither antibody in B.1.1.7.  2. The B.1.351 and B.1.1.28 lineages contain combinations of mutations that escape each component of the LY-CoV555+LY-CoV016 cocktail.  3. LY-CoV555+LY-CoV016 cocktail may be ineffective against these lineages |
| Wang P, et al[45] | mRNA-1273(Moderna)+ BNT162b2(Pfizer)  mRNA Vaccine | B.1.1.28 pseudovirus (2.8 fold, Moderna; 2.2 fold, Pfizer) and B.1.351 pseudovirus (8.6 fold, Moderna; 6.5 fold, Pfizer) drop in neutralizing titers. |
| Wang P, et al[44] | mRNA-1273(Moderna)+ BNT162b2(Pfizer)+ 12 RBD mAbs  mRNA Vaccine+mAb | 1. B.1.1.7 is refractory to neutralization by most mAbs to the N-terminal domain (NTD) of the spike and relatively resistant to a few mAbs to RBD.  2. B.1.351 is markedly more resistant to neutralization by convalescent plasma (9.4 fold) and vaccinee sera (10.3-12.4 fold). |
| Collier DA, et al[46] | BNT162b2(Pfizer)  mRNA Vaccine | 1. The vaccine sera and convalescent sera exhibited modestly reduced neutralising titres against B.1.1.7 variant.  2. Introduction of the E484K mutation in a B.1.1.7 background led to a more substantial loss of neutralising activity by vaccine-elicited antibodies and mAbs (19 out of 31) over that conferred by the B.1.1.7 mutations alone. |

Table B Characteristics of New Variants

| New variants | Transmissibility | Antigenicity | Severity | Vaccination | Treatment | Zoonotic emergence | Cross-border spread |
| --- | --- | --- | --- | --- | --- | --- | --- |
| B.1.1.7 | Increasing | Increasing | Unclear | Unclear | Unclear | Unclear | Yes |
| B.1.351 | Increasing | Increasing | Unclear | Likely influenced | Likely influenced | Unclear | Yes |
| P.1 | Increasing | Increasing | Unclear | Likely influenced | Likely influenced | Unclear | Yes |
| B.1.525 | Increasing | Unclear | Unclear | Unclear | Unclear | Unclear | Unclear |
| CAL.20C | Increasing | Unclear | Unclear | Unclear | Unclear | Unclear | Unclear |
| COH.20G | Unclear | Unclear | Unclear | Unclear | Unclear | Unclear | Unclear |
| Cluster 5 | Unclear | Unclear | Unclear | Unclear | Unclear | Yes, Mink | Yes |
| B.1.1.207 | Unclear | Unclear | Unclear | Unclear | Unclear | Unclear | Unclear |
